# *In silico* phenotype projection of endothelial ERK1/2 signaling

**DOI:** 10.18632/aging.103529

**Published:** 2020-06-12

**Authors:** Michael Simons, Jeffrey R. Gulcher, Thomas W. Chittenden

**Affiliations:** 1 Yale Cardiovascular Research Center, New Haven, CT 06510, USA; 2 Department of Cell Biology, Yale University School of Medicine, New Haven, CT 06510, USA; 3 WuXi NextCode Inc., Cambridge, MA 02142, USA; 4 Divison of Genetics and Genomics, Boston Children’s Hospital, Harvard Medical School, Boston, MA 02115, USA

**Keywords:** hypertension, vascular regulation, endothelial-to-mesenchymal transition, EndMT, TGFβ signaling, probabilistic programming, phenotype projection

One of the challenges of modern-day biology is a rapid and cost-effective functional evaluation of novel (or poorly understood) genes. Typically, *in vitro* approaches are first used to alter gene expression using various RNAi and/or transfection approaches. This is followed by an array of cell physiology assays and signaling studies that can examine cell proliferation, migration, adhesion, cytoskeletal organization, responses to specific agonists or metabolic perturbations, among others. Frequently, the data generated are not sufficient to have a clear idea about molecular pathways involved and potential physiological impacts. Therefore, these studies are then followed by *in vivo* evaluations using organisms such as mice and zebrafish. The process of creating genetically engineered animals with altered gene expression followed by phenotype evaluation is both time- and resource-consuming and functional alterations may be hard to pinpoint. Thus, there is a strong need for an alternative strategy that would predict functional outcomes based on *in silico* gene expression profiling.

The advent of inexpensive sequencing techniques, including bulk DNA, bulk RNA and single cell RNA sequencing makes such an approach feasible. Yet there remains a challenge of interpreting these large-scale omics data and making functional predictions. An important step forward in this regard has been the development of an ensemble artificial intelligence strategy that combines integrated deep learning and probabilistic programming. This causal statistical computing framework is designed to generate working hypotheses from high-dimensional datasets without the use of *a priori* biological knowledge. This strategy affords the derivation of highly informative putative causal dependency structures within gene interaction networks based on conditional probabilities of gene expression states. The goal is to discern hierarchical relationships driving changes in gene expression between two or more different states. In its simplest form it can be used to unveil genetic relationship networks following exposure of a cell population to a signal. For example, to predict the role of gene X in a particular cell type, this computing framework strategy can be used to analyze changes in gene expression before and after gene X knockout or overexpression in order to arrive at a functional role played by this gene.

This statistical computing framework was tested in the context of extracellular response kinase (ERK) signaling in endothelial cells. ERKs are a part of a ubiquitous and extensive family of mitogen-activated kinases (MAPK) that are involved in numerous biological processes and orchestrate a complex series of biological events [1,2]. While the overall pattern of MAPK/ERK signaling is well understood, specific roles played by individual members in various cell types have proved surprisingly hard to pinpoint [3]. This gene family thus presents an ideal proof of concept for AI-driven biological discovery. The test was designed around analysis of 20 independent bulk RNA-seq preparations of human endothelial cells subjected to ERK1/2 siRNA knockdown or treatment with a control siRNA sequence. Sixteen pairs of data were used for *in silico* analyses, while 4 were then used for functional predictions [4].

**Figure 1:**
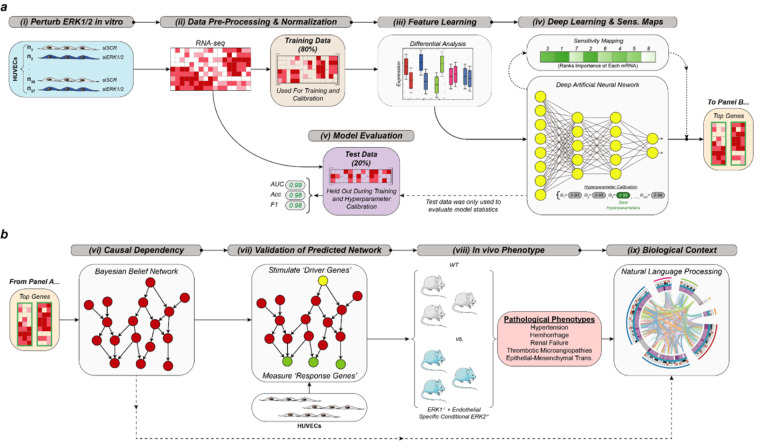
**Overview of statistical computing framework for in silico phenotype projection.** (**a**) experimental perturbation of ERK1/2 in human umbilical vein endothelial cells (HUVEC), data processing, feature learning, deep learning, and statistical assessment of model classification performance of single cell RNA-seq data. (**b**) Bayesian Belief Network analysis (BBN), experimental validation of putative causal gene dependency structure in HUVEC, experimental evaluation of predicted phenotypes in mice, and natural language processing of results. Modified from Figure S5, Ricard et al., JEM 2019.

After initial data processing and feature learning to reduce dimensionality of the analysis, a deep artificial neural network (DANN) was trained and tested to predict experimental and control endothelial cell conditions based on gene expression profiles. The most informative genes identified by the DANN model were then used to build as Bayesian belief network (BBN). The BBN produced a putative causal gene dependency structure, which provided biological context for all downstream analyses (Figure 1). For example, the subsequent directed acyclic graph (DAG) of the BBN was comprised of driver and responder genes linked by a number of intermediary drivers and responders. Quantitative assessment of the degree of node connectivity within DAG revealed activation of transforming growth factor beta (TGFβ) signaling as one of the main drivers of the gene interaction network. Endothelial TGFβ signaling has been previously linked to a cell fate change termed endothelial-to-mesenchymal transition (EndMT) [5,6]. In agreement with this finding, the DAG causally linked numerous EndMT genes including genes associated with tissue fibrosis and, in particular, renal fibrosis to endothelial TGFβ signaling. Another set of findings was a decrease in expression of NOS3 that produces a vasodilator nitric oxide and an increase in expression of a vasoconstrictive hormone endothelin 1. Together, these results pointed to likely development of hypertension.

These predictions were tested in mice with an endothelial-specific disruption of ERK signaling. Phenotypic and functional evaluation revealed that following deletion of ERK1/2 genes in adult endothelial cells, mice developed hypertension due to a decrease in NO production (driven by reduced NOS3 expression) that was followed, at a later time point, by increased endothelin-1 expression [4]. Along with hypertension, there was extensive EndMT and fibrosis in multiple organs, findings consistent with activation of TGFβ signaling [7], including progressive renal failure due to a TGFβ-driven glomerulonephropathy [8]. These findings, therefore, indicate a close agreement between AI-based predictions based on an in vitro RNAseq data set and in vivo biology.

This study thus provides the first biologically validated evidence that intelligently engineered machine learning strategies can glean meaningful insights into highly complex disease etiologies by robustly inferring informative gene network dependencies, reflective of the signal transduction cascades that drive cellular behavior and dictate phenotype. Analogous to established experimental methods of reverse genetics, this *in silico* phenotype projection is a highly efficient AI-driven approach capable of predicting complex phenotypes by teasing out the causal molecular underpinnings of disease. While requiring further validation, these results point to the significant potential for AI-driven analysis of omics data.

Our findings suggest that asymmetric divisions are the predominant mode of stem cell division in the adult hippocampus. This calls for reevaluating widely held assumptions on the distribution, maintenance, and divisions of neural stem cells, on the possibility of switching modes of division in response to cognitive or emotional stimuli, environment, and disease, and on the prospects of rejuvenation of the ailing nervous system. These findings also emphasize the need for finding new agents and targets for controlling the modes of stem cell division: if neural stem cells are not dividing symmetrically on their own, then we may need to induce that mode of replication to repair the aging or damaged brain.
